# Serum cystatin C was a marker of poststroke fatigue in hypertensive intracerebral hemorrhage

**DOI:** 10.1002/brb3.1969

**Published:** 2020-11-26

**Authors:** Fulan Yang, Peipei Liu, Saiyu Huang, Xiaojie Liu, Xue Gao, Chunyin Liu, Lanlan Chen, Yingzhu Chen

**Affiliations:** ^1^ Department of Neurology Chongqing Emergency Medical Center Chongqing University Chongqing China; ^2^ Department of Neurology Clinical Medical College Northern Jiangsu People’s Hospital Yangzhou University Yangzhou China; ^3^ Department of Neurology The People’s Hospital of Bozhou Bozhou China

**Keywords:** cystatin C, hypertensive intracerebral hemorrhage, poststroke fatigue, stroke

## Abstract

**Introduction:**

The relationship between poststroke fatigue (PSF) and serum Cystatin C (Cys‐C) levels in hypertensive intracerebral hemorrhage (HICH) patients has not been determined. In this study, we investigated the association between serum Cys‐C levels and PSF in HICH patients.

**Methods:**

A total of 125 patients with HICH were enrolled. Fatigue assessment was performed 6 months after HICH onset. The presence of PSF was defined as Fatigue Severity Scale (FSS) of 4 or more. Serum Cys‐C levels were measured within 24 hr after admission. The correlation between FSS score and Cys‐C level was analyzed by Spearman's correlation. Receiver operating characteristic (ROC) curves for PSF were calculated using Cys‐C values.

**Results:**

Of enrolled 125 patients in the study, 36.0% who developed PSF were divided to the PSF group, which had higher Cys‐C levels compared with the no‐PSF group. There was significant positive correlation between FSS score and serum Cys‐C level. Receiver operating characteristic curves for PSF revealed an area under the curve of 0.86 for Cys‐C. High admission Cys‐C (>0.75mg/L) yielded specificity of 93.7%, positive predictive value of 87.5%, and negative predictive value of 88.2%. In multivariate analysis, Cys‐C increased by 1 mg/dl (0.1 mg/L), and the risk of PSF in patients increased by 2.55 times (odds ratio = 2.55, 95% CI: 1.65–3.95, *p* < .001).

**Conclusions:**

High Cys‐C levels have predictive value for PSF and can be used as one screening indicator for PSF occurrence.

## Graphical abstract

High Cys‐C levels have predictive value for PSF and can be used as one screening indicator for PSF occurrence. High admission Cys‐C (>0.75 mg/L) yielded specificity of 93.7%, positive predictive value of 87.5%, and negative predictive value of 88.2%.

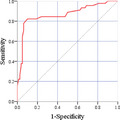

## 1 INTRODUCTION

Poststroke fatigue (PSF) was defined PSF as fatigue and exhaustion that can occur shortly after stroke or prematurely after long‐term physical and mental activity, which usually cannot be relieved after a break (Glader et al., 2002). PSF represents a complex biological interaction, which is often a chronic and persistent psychosocial and behavioral phenomenon (Barker‐Collo et al., 2007), and gradually gained more attention in recent years (Leegaard, 1983). PSF is a common problem among stroke survivors, and the frequencies of PSF were reported from 25% to 85% (Cumming et al., 2016). Fatigue impaired the patient's ability to regain functions after stroke, decreased the quality of life, and was related to the long‐term mortality rate (Glader et al., 2002). How to prevent the occurrence of PSF is important for improving the quality of life of patients after stroke.

Previous studies on PSF have mostly focused on patients with ischemic stroke. However, regarding pathological type of stroke and fatigue, few studies have concluded that fatigue is more severe after ischemic stroke than after intracerebral hemorrhage (Naess et al., 2010). In terms of incidence of PSF, study even found that a trend (*p *= .076) toward higher fatigue after hemorrhagic stroke (69%) than ischemic stroke (49%) (Cumming et al., 2016).

Cystatin C (Cys‐C), a low‐molecular weight inhibitor of cysteine protease which was produced and released at a constant rate from all nucleated cells (Tang et al., 2008), is a more sensitive indicator of renal function compared to serum creatinine and glomerular filtration rate (Dharnidharka et al., 2002). Recent years, many studies have found that Cys‐C was closely related to cardiovascular and cerebrovascular diseases (Gevorgyan et al., 2017; Zhang & Sun, 2017). Serum Cys‐C levels in patients with intracerebral hemorrhage were significantly higher than those in hypertensive patients and healthy people, and Cys‐C levels in stroke patients were positively correlated with lesion volume (Xiao et al., 2012). It was also found that the development of PSF was associated with basal ganglia infarcts (Tang et al., 2010). Hypertensive intracerebral hemorrhage (HICH) occurs mostly in the basal ganglia due to anatomical reasons. Serum Cys‐C, which was reported as an independent risk factor for cerebral hemorrhage (Xiao et al., 2012), may play an important role in the prognosis of HICH and the occurrence of PSF.

However, as far as we know, none study till now had determined the relationship between PSF and the Cys‐C level in HICH. The aim of this study was to assess the association between PSF and Cys‐C level in HICH patients. We hypothesized that a series of changes after cerebral hemorrhage lead to PSF, which will also affect the kidneys, resulting in an increase in serum Cys‐C levels. We tested this hypothesis in a cohort of prospectively ascertained patients.

## 2 METHODS

### 2.1 Study population

Participants were recruited from patients consecutively admitted with first‐ever HICH to the Department of Neurology of Northern Jiangsu People's Hospital from October 2017 to December 2018.

The inclusion criteria for the study were as follows: (a) well‐documented (clinical presentation and CT/MRI scan of the brain) first spontaneous cerebral hemorrhage; (b) the onset time at admission within 24 hr; (c) vital signs were stable; (d) age of 18 years or above; and (e) obtain approval from the ethics committee and informed consent of the patient.

The exclusion criteria included the following: (a) secondary cerebral hemorrhage caused by trauma, intracranial vascular malformation, cerebral aneurysm, intracranial space, etc.; (b) history of stroke or severe intracranial organic disease, encephalitis, multiple sclerosis, Parkinson's disease, cancer, systemic lupus erythematosus, blood disease, severe liver and kidney dysfunction, severe heart disease, metabolic disease (except diabetes), and infectious and inflammatory diseases; (c) patients selected to undergoing surgery; (d) moderate and severe cognitive impairment defined by a mini‐mental state examination (MMSE) score of less than 20 (Rosen, 1996); (e) aphasia; and (f) Prestroke fatigue (PrSF). PrSF was measured retrospective by two items: “did you experience fatigue before you had your stroke” (yes/no) and if yes, “how long did you experience fatigue.” Patients who reported feeling fatigue for more than 3 months before the onset of stroke were defined as having PrSF (Lerdal et al., 2011); (g) poststroke depression, two trained neurologists administered the Chinese version of the structured clinical interview for DSM‐IV to made the diagnosis of depression (Hu, 2003); (h) patients with stroke or other serious illnesses or even death occurred again during the 6‐month follow‐up period.

Ethical approval was obtained from the Ethics Committee of the Northern Jiangsu People's Hospital. All investigations were conducted in accordance with the principles expressed in the Declaration of Helsinki. Written informed consent was obtained from all participants. All personal information was encrypted.

### 2.2 Collection of demographic and clinical data

The baseline clinical and demographic information, including age, sex, systolic blood pressure (SBP), diastolic blood pressure (DBP), history of diabetes mellitus, history of smoking, history of drinking, and National Institute of Health Stroke Scale (NIHSS), was recorded after admission. Admission laboratory data, including Cys‐C, urea nitrogen, creatinine, uric acid, triglycerides, high‐density lipoprotein cholesterol, low‐density lipoprotein cholesterol, and total cholesterol, also were recorded. The serum Cys‐C level was measured using an enzyme‐labeled method on a Hitachi 7170 automatic biochemical analysis (Hitachi).

Fasting blood was exsanguinated from the vein by medical professionals within 24 hr after admission. Blood samples were then sent to the department of Clinical Laboratory, Northern Jiangsu People's Hospital, and measured on automated instruments.

### 2.3 Assessment of PSF

The interviews were conducted 6 months after the stroke through in‐person interview or telephone interview. The researcher administered the Chinese version of the fatigue severity scale (FSS) (Schwartz et al., 1993) which had previously been used to evaluate fatigue in stroke (Choi‐Kwon et al., (2007); Schepers et al., 2006; Delva et al., 2018). It contains nine seven‐point Likert items, with a higher score indicating more fatigue. The answers were then averaged, resulting in overall fatigue scores. The presence of PSF was defined as FSS scores of 4 or more.

At the 6‐month follow‐up, the 24‐item Hamilton Depression Scale (HAMD‐24) (Hamilton, 1981) and 4‐item Hamilton Anxiety Scale (HAMA‐14) (Maier et al., 1988) were used to measure depression and anxiety symptoms, respectively (Moonen et al., 2014), and the 9‐item version of FSS and the modified Rankin Scale (mRS) score were administered to assess the participants’ fatigue symptoms and functional outcome, respectively. Poor outcome was defined as mRS > 2, and good outcome was defined as mRS ≤ 2.

### 2.4 Statistical analysis

The participants were divided into no‐PSF and PSF groups according to FSS score. We compared continuous variables using Student's *t* tests for normally distributed variables or Mann–Whitney *U* tests for non‐normally distributed variables. Categorical variables were compared using chi‐square tests, depending on the expected cell counts. The correlation between FSS score and Cys‐C level was analyzed using Spearman's correlation. Receiver operating characteristic (ROC) curves for PSF were calculated using Cys‐C values. The area under the curve (AUC) was calculated, and based on the ROC curves, best‐performing cutoff was selected. Multivariate logistic regression analysis was performed to investigate the effects on Cys‐C and PSF as a dependent variable. Sensitivity, specificity, positive predictive value, and negative predictive value were calculated for PSF. Statistical analyses were undertaken using SPSS version 22.0 software (SPSS). A *p* value < .05 was considered statistically significant.

## 3 RESULTS

### 3.1 Demographic and clinical characteristics

A total of 332 patients were included in the study. During the 6‐month follow‐up period, 8 patients died, 34 had an onset more than 24 hr, 63 had a history of stroke, 22 suffered from tumors, severe liver dysfunction and other diseases, 26 selected to undergoing surgery, 39 were diagnosed with PrSF or PSD, and 15 were lost to follow‐up. There were no differences in baseline characteristics between those included in and excluded from the final analysis. As a result, data from 125 patients were available for analysis.

Among the 125 enrolled HICH patients, 45 (36.0%) were diagnosed with PSF and 80 (64.0%) had no PSF, respectively. Characteristics of patients were shown in Table 1. Compared with the no‐PSF group, the PSF group was more likely to be female and elder and had higher total cholesterol and higher Cys‐C level at admission, with higher NIHSS score at admission, higher HAMD score at 6‐month, lower MMSE score at 6‐month, and worse outcome at 6‐month. There were no significant differences in other clinical features between the groups.

**Table 1:** Clinical characteristics of the participants according to PSF status

Characteristics	no‐PSF (*n* = 80)	PSF (*n* = 45)	*p* value
Age, years^a^	60.00 (48.00–68.00)	65.00 (54.50–73.50)	.035^b^
Sex, female, *n* (%)	26 (32.50)	23 (51.11)	.041^c^
Admission SBP, mmHg^a^	160.00 (146.00–178.00)	164.00 (140.00–180.00)	.623^b^
Admission DBP, mmHg^a^	90.00 (80.00–98.00)	90.00 (76.50–104.00)	.648^b^
Diabetes mellitus, *n* (%)	8 (10.00)	8 (17.78)	.212^c^
Blood glucose, mmol/L^a^	5.70 (5.04–6.60)	5.89 (5.36–7.16)	.406^d^
Tobacco consumption, *n* (%)	27 (33.75)	8 (17.78)	.056^c^
Alcohol consumption, *n* (%)	22 (27.50)	10 (22.22)	.516^c^
Total cholesterol, mmol/L^a^	4.42 (3.88–5.10)	4.95 (4.13–5.48)	.022^b^
Triglycerides, mmol/L^a^	1.36 (0.96–1.27)	1.28 (1.01–1.87)	.907^d^
LDL‐C, mmol/L^a^	2.62 (2.17–3.12)	2.72 (2.17–3.19)	.768^b^
HDL‐C, mmol/L^a^	1.27 (0.96–1.56)	1.41 (1.11–1.66)	.068^b^
Urea nitrogen, mmol/L^a^	5.39 (4.30–6.00)	5.53 (4.57–6.57)	.220^b^
Creatinine, umol/L^a^	64.00 (54.00–76.00)	63.00 (53.50–74.50)	.717^d^
Uric acid, umol/L^a^	312.00 (247.00–371.00)	298.00 (248.50–377.50)	.643^d^
Cys‐C, mg/L^a^	0.60 (0.50–0.70)	0.90 (0.76–1.02)	<.001^d^
NIHSS score^a^	5.00 (3.00–13.00)	12.00 (6.50–16.50)	<.001^d^
mRS > 2, *n* (%)	8 (10.00)	24 (53.33)	<.001^c^
MMSE score^a^	27.00 (25.00–28.00)	25.00 (24.00–28.00)	.023^d^
HAMA score^a^	11.00 (9.00–15.00)	13.00 (9.50–19.00)	.084^d^
HAMD score^a^	4.00 (2.00–6.00)	6.00 (5.00–8.00)	<.001^d^

Abbreviations: Cys‐C, Cystatin C; HAMA, Hamilton Anxiety Scale; HAMD, Hamilton Depression Scale; HDL‐C, high‐density lipoprotein cholesterol; LDL‐C, low‐density lipoprotein cholesterol; MMSE, mini‐mental state examination; mRS, modified Rankin Scale; NIHSS, National Institute of Health Stroke Scale; PSF, poststroke fatigue.

^a^ median (interquartile range).

^b^ *t* test.

^c^ chi‐square tests.

^d^ Mann–Whitney *U* test.

### 3.2 Association between PSF and Cys‐C

FSS score was positively correlated with Cys‐C level in the total 125 patients and the PSP group. However, there was no correlation between FSS score and Cys‐C in the non‐PSF group (Table 2).

**Table 2:** Correlations between FSS score and serum Cys‐C level

	*r*	*p* value
total patients	0.58	.001
PSF group	0.46	.001
non‐PSF group	0.08	.452

Abbreviation: PSF, poststroke fatigue.

### 3.3 ROC curves analysis of predicting PSF by Cys‐C

**Figure 1:**
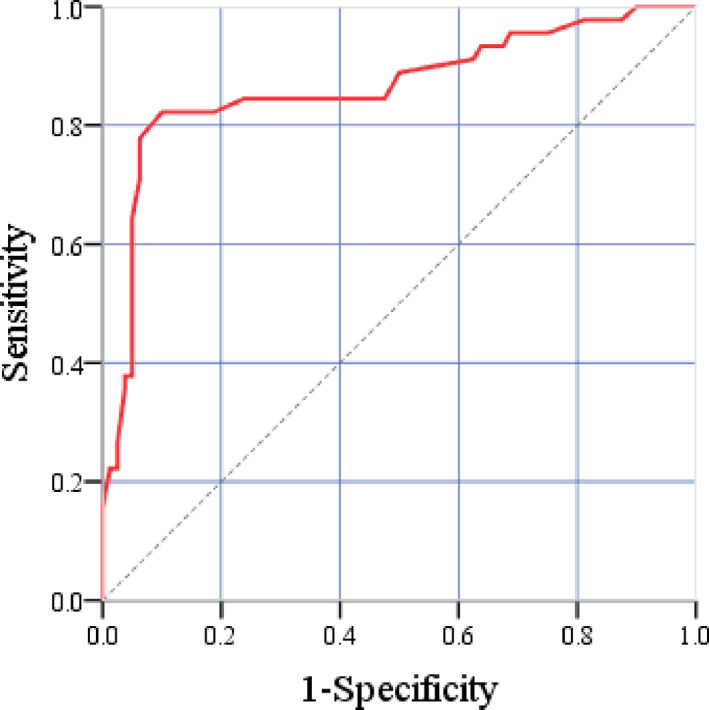
ROC curves of predicting PSF with serum Cys‐C level

ROC curves yielded an AUC of 0.86 (95% CI: 0.79–0.94) for Cys‐C. The optimal cutoff value was 0.75 mg/L of Cys‐C with the sensitivity and specificity in predicting PSF in patients with HICH were 77.8% and 93.7%, respectively (Figure 1).

### 3.4 The predicting value of optimal cutoff value of Cys‐C for PSF

In univariate analysis, Cys‐C > 0.75 mg/L showed high specificity of 93.7%, positive predictive value of 87.5%, and negative predictive value of 88.2% as predictive tests for the development of PSF (Table 3).

**Table 3:** Sensitivity, specificity, positive predictive value, and negative predictive value of Cys‐C as predictive tests for PSF

	Sensitivity (95% CI)	Specificity (95% CI)	PPV (95% CI)	NPV (95% CI)
Cys‐C > 0.75 mg/L	77.8% (65.15–90.41)	93.7% (88.33–99.17)	87.5% (76.79–98.21)	88.2% (81.24–95.23)

Abbreviations: CI, confidence interval;Cys‐C, Cystatin C; NPV, negative predictive value; PPV, positive predictive value.

### 3.5 Multivariate logistic regression between PSF and risk factors

In view of account the small molecular weight of cys‐C, it is difficult to increase 1 mg/L, so we set the unit of cystatin C to mg/dl, and then took it into the multivariate logistic regression analysis model together with age, gender, total cholesterol, NIHSS score, mRS > 2, MMSE score, and HAMD score. Table 4 showed that Cys‐C, mRS > 2, and HAMD score were significant independent predictors of PSF. And Cys‐C increased by 1 mg/dl (0.1 mg/L), and the risk of PSF in patients increased by 2.55 times (odds ratio = 2.55, 95% CI: 1.65–3.95, *p* < .001) (Table 4).

**Table 4:** Predictors for PSF estimated by multivariate logistic analysis model

Factor	*β*	OR	*p* value	95% CI
Cys‐C, mg/dl	0.94	2.55	<.001	1.65–3.95
mRS > 2	2.45	11.60	.002	2.43–55.42
HAMD score	0.45	1.57	<.001	1.24–1.99

Abbreviations: CI, confidence interval; Cys‐C, Cystatin C; HAMD, Hamilton Depression Scale; mRS, modified Rankin Scale; OR, odds ratio.

## 4 DISCUSSION

PSF, a complication in the recovery process of stroke, will lower the quality of life of patients and is related to the long‐term mortality rate. Previous research has mainly focused on the effects of psychological factors, depression, and cognitive abilities in poststroke patients on the incidence of PSF, and there were fewer studies on biochemical indicators (Annoni et al., 2008). In this study, we investigated the relationship between Cys‐C and PSF in HICH patients. The results suggested that PSF was associated with the serum Cys‐C level. Specifically, an increased Cys‐C level was shown to result in a corresponding increase in the risk of occurrence of PSF, even after been adjusted the confounding factors.

The mechanism of PSF has not yet been elucidated. Chaudhuri and Behan (Chaudhuri & Behan, 2004) believed that fatigue was a feeling that it was difficult to initiate or maintain voluntary activities. Voluntary activities are regulated by biological systems in the body. Destruction of the system will cause fatigue, and homeostasis imbalances (such as sleep disorders) can also cause fatigue. Study by Kuppuswamy et al. (2014) had shown that reduced excitability of the input signal in the cortical and subcortical facilitation regions or decreased excitability of the output signal in the cortical medulla may result in heavier PSF. Therefore, a decrease in neuronal activity after stroke reduces the excitability of the neuron, which in turn produces a feeling of fatigue.

A meta‐analysis which identified factors associated with PSF reported that PSF associated with emotional disturbances, pain, sleep disorders, and PrSF (Ponchel et al., 2015). Sarfo et al. (2019) demonstrated that age ≥ 65 years, female, and depression remained significantly associated with PSF at 9 months among Ghanaians. Our results also demonstrated that older age, female sex, and high HADM score were significantly associated with PSF (Table 1). Study concluded that fatigue was associated with poor functional outcome (mRS > 2) in transient ischemic attack and ischemic stroke patients (Maaijwee et al., 2015). Our study found that this conclusion was equally applicable to HICH patients. The results showed that HADM score was an independent predictor of PSF even after excluding the poststroke depression in this study, suggesting that depression was closely related to fatigue (Tang et al., (2010)). Interestingly, we also found that Cys‐C increased by 1 mg/dl (0.1 mg/L), and the risk of PSF in patients increased by 2.55 times (odds ratio = 2.55, 95% CI: 1.65–3.95, *p* < .001).

Few studies had explored the association between the Cys‐C and PSF. Cys‐C, as one of the well‐known widely distributed cathepsin inhibitors, exists in all body fluids and participates in and regulates many physiological and pathological processes, including cell proliferation, inflammation response, antibacterial, tumor metastasis, and bone matrix reabsorption (Reed, 2000). Cys‐C was involved in the damage and repair of neuronal tissues in the brain (Peng et al., (2019)). Studies demonstrated high serum Cys‐C level was independently associated with both ischemic stroke and cerebral hemorrhage, and Cys‐C level was an independent predictor for the risk of cardiovascular events and death in stroke patients (Xiao et al., (2012); You et al., 2016; Ni et al., 2007; Wang et al., 2019). Zeng et al. (2019) found that Cys‐C was significantly associated with vascular cognitive impairment.

Mechanisms underlying the relationship between high Cys‐C and stroke have been reported as follows. First, Cys‐C is involved in the pathophysiological process of atherosclerosis (Zeng et al., (2015); Xu et al., 2017). Second, a large amount of Cys‐C in the cerebrospinal fluid enters the blood circulation through the pathologically blood–brain barrier at stroke onset. Third, as an inhibitor of cysteine proteases, high concentration of serum Cys‐C affects the process of vascular wall remodeling by breaking the balance of proteolytic and antiproteolytic activities (Liu et al., 2004). In addition, it was found that acute phase serum high glucose, high interleukin‐1β, and low interleukin‐9 can predict PSF, suggesting that PSF can occur through a related proinflammatory response. These new findings support the new cytokine theory of PSF (Ormstad et al., 2011). Cys‐C could affect the migration of neutrophils, and its fragments may also affect the phagocytic and chemotactic functions of granulocytes and participate in the inflammatory process (Hanrieder et al., 2009). This may be one of the reasons for elevated serum Cys‐C levels in patients with PSF.

The diagnosis and evaluation of PSF usually requires the use of related scales, such as the FSS, Fatigue Impact Scale (FIS), and Fatigue Assessment Scale (FAS). These scales usually take a lot of time to complete the questionnaire, fill in forms and calculate the score. Compared with the more cumbersome scale scoring system in routine clinical practice, the high specificity, PPV, and NPV of serum Cys‐C levels for the development of PSF may serve as a screening indicator.

There are some limitations in this study. First, the sample size was relatively small. Second, due to the wide variety of scales for evaluating PSF, the focus is different. We just selected the FSS scale mainly reflecting the physical fatigue of the body but little evaluation of the psychological fatigue of cognitive and emotional factors of fatigue patients. In the future, more comprehensive scales will be used to predict the factors associated with the PSF accurately. Despite these limitations, this study had some advantages. This was the first study to explore the correlation between Cys‐C and PSF in HICH patients. In addition, the results of this study suggested a potential role for the Cys‐C in the pathogenesis of PSF in HICH patients.

In conclusion, this study demonstrated that increased serum Cys‐C level was strongly associated with PSF in patients with HICH. Thus, detection of the serum Cys‐C level might be valuable for early screening individuals at risk of PSF. Our findings provided a new evidence connecting PSF and Cys‐C in patients with HICH, and lots of clinical research are needed for further verification.

## CONFLICTS OF INTEREST

The authors declare no competing financial interests.

## AUTHOR CONTRIBUTIONS

Fulan Yang, Peipei Liu and Saiyu Huang contributed equally to the study as the cofirst authors. FLY, PPL, SYH, XJL, LLC, and YZC were responsible for the study concept and design. FLY, XG, and CYL involved in collection of data and analysis and interpretation. FLY, PPL, and SYH wrote the first draft. YZC and LLC involved in review and critique. All authors were responsible for a critical revision of the manuscript for important intellectual content. YZC and LLC were the study supervisor.

### Peer Review

The peer review history for this article is available at https://publons.com/publon/10.1002/brb3.1969.

## Data Availability

The data of our study will be available via connecting with Dr. yingzhu Chen (corresponding author).
